# The Effects of Different Types of Steroids on Clinical Outcomes in Neonates with Meconium Aspiration Syndrome: A Systematic Review, Meta-Analysis and GRADE Assessment

**DOI:** 10.3390/medicina57111281

**Published:** 2021-11-21

**Authors:** Nanthida Phattraprayoon, Teerapat Ungtrakul, Wimonchat Tangamornsuksan

**Affiliations:** 1 Faculty of Medicine and Public Health, HRH Princess Chulabhorn College of Medical Science, Chulabhorn Royal Academy, Bangkok 10210, Thailand; nanthida.pha@cra.ac.th (N.P.); teerapat.ung@cra.ac.th (T.U.); 2 Department of Health Research Methods, Evidence and Impact, McMaster University, Hamilton, ON L8S 4L8, Canada

**Keywords:** different types of steroids, meconium aspiration syndrome, clinical outcomes, systematic review and meta-analysis

## Abstract

*Background and Objectives*: Meconium aspiration syndrome (MAS) is a condition caused by the aspiration of meconium-stainted amniotic fluid into the lungs, resulting in pulmonary inflammation, neonatal morbidity, and mortality. It is important that these MAS infants receive appropriate care to avoid further complications. Steroids have an anti-inflammatory effect and may be effective in the management of MAS. The objective of the this study was to evaluate the effect of different steroids on clinical outcomes in infants with MAS. *Materials and Methods*: We systematically searched of PubMed/Medline, Scopus, Embase, Clinical Trials.gov, and Cochrane Library databases from inception to 24 January 2021 without language restriction. Only randomized controlled trials (RCTs) evaluating the effects of steroids in neonates with MAS were included. We calculated relative risks and weighted mean differences (MDs) with 95% confidence intervals (CIs) using a random-effects model to determine the associations between MAS and steroids and GRADE approach was employed for quality of evidence. The main outcomes measures were duration of respiratory distress, oxygen requirement, hospitalization, need for mechanical ventilation, death, and adverse drug reactions. *Results*: Seven RCTs involving 397 patients were analyzed. Nebulized budesonide and intravenous (IV) methylprednisolone shortened the duration of respiratory distress (MD, −2.46 days; 95% CI, −3.09 to −1.83 and MD, −3.30 days; 95% CI, −4.07 to −2.52, respectively) (moderate certainty). There was a reduction in duration of oxygen requirement in nebulized budesonide use (MD, −2.40 days; 95% CI, −3.40 to −1.40) (low certainty) and IV methylprednisolone use (MD, −3.30 days; 95% CI, −4.07 to −2.52) (moderate certainty). Nebulized budesonide shortened hospitalization stay (MD, −4.47 days; 95% CI, −8.64 to −0.30 days) (low certainty) as IV methylprednisolone use (MD, −7.23 days; 95% CI, −8.19 to −6.07 days) (moderate certainty). None of steroids benefits in death (low certainty). *Conclusions*: Certain types of steroids may benefit the respiratory aspect, but there was no decrease in mortality in MAS infants.

## 1. Introduction

Meconium aspiration syndrome (MAS) is caused by aspiration of meconium-containing amniotic fluid into the lungs. The complex chemical composition of meconium may be responsible for pulmonary inflammation, termed chemical pneumonitis, and a risk of surfactant inactivation [1]. The aspirated meconium may also cause mechanical obstruction of small airways. In neonates with partial airway occlusion, the over-expanded lungs will increase air leakage complications [2]. Around 5% to 20% of meconium-stained amniotic fluid (MSAF) infants develop MAS [3,4], with mortality rate of 5% [5,6]. The incidence of MAS is declining in developed countries with advances in obstetric practices and perinatal care [7,8], however, MAS remains a significant respiratory problem and cause of neonatal mortality in developing countries [9,10]. Management of MAS mainly involves supportive care to ensure appropriate oxygenation and ventilation, including stabilization of the systemic circulation, in an effort to prevent other serious conditions such as persistent pulmonary hypertension of the newborn (PPHN) [11]. The pathophysiology of MAS depends on the severity of inflammation, and hence suppressing inflammation may improve clinical outcomes in MAS infants. Steroids can down-regulate proinflammatory cytokine production in vitro [12,13,14]. Using steroids to suppress inflammation in infants with MAS may also be beneficial. However, there is a lack of sufficient evidence to assess the potential benefits and detriments of the use of steroids in MAS [15]. Therefore, we conducted a systematic review and meta-analysis of the efficacy of different types of steroids in MAS.

## 2. Materials and Methods

The study protocol was registered in PROSPERO (registration number CRD42020211334). This systematic review and meta-analysis was performed according to the Preferred Reporting Items for Systematic Reviews and Meta-Analyses (PRISMA).

The main outcomes were the duration of respiratory distress, oxygen requirement, hospitalization, need for mechanical ventilation, death, complications and adverse drug reactions (ADRs).

### 2.1. Search Strategy and Selection Criteria

We performed a comprehensive and systematic search of the PubMed/Medline (U.S. National Library of Medicine, Bethesda, MD, USA), Scopus, Embase, Clinical Trials.gov, and Cochrane Library databases from inception to 24 January 2021 using keywords, synonyms, and other terms related to MAS and steroids without language restriction. Only randomized controlled trials (RCTs) were included. Additional studies were identified via the reference lists of selected articles.

Two reviewers (N.P. and W.T.) separately and independently screened and selected studies using the eligibility criteria. Any disagreements were resolved by discussion with a third reviewer (T.U.).

### 2.2. Data Extraction and Risk-of-Bias (Quality) Assessment

Two reviewers (N.P. and W.T.) separately extracted data from the included studies, including the study design and methodology, eligibility and diagnostic criteria, patient demographics, data collection method, definition of outcomes and outcomes parameters, and number of events. Study investigators were contacted for any missing data, unreported data, and additional details.

Two reviewers (N.P. and W.T.) independently evaluated the quality of the included studies using the Revised Cochrane risk-of-bias tool for randomized trials (RoB 2) [16]. Any disagreements were resolved by discussion with a third reviewer (T.U.).

### 2.3. Data Analyses

We calculated relative risks (RRs) and weighted mean differences (MDs) with 95% confidence intervals (CIs) using the DerSimonian and Laird method with a random-effects model to determine the associations between MAS and steroids in neonates with MAS for dichotomous and continuous outcomes, respectively [17]. We performed a separate analysis based on the type of steroids and assessed statistical heterogeneity via Q-statistic and I² tests. *p*-values of ≤0.05 indicated heterogeneity between studies [18]. I² values of 25%, 50%, and 75% denoted low, moderate, and high heterogeneity across studies, respectively [19]. If ≥10 studies proved eligible in each outcome, publication bias was evaluated using a funnel plot [20]. All statistical analyses were performed using Stata version 16.0 (StataCorp LLC, College Station, TX, USA).

### 2.4. Quality of Evidence

We used the Grading of Recommendations Assessment, Development and Evaluation (GRADE) approach to rate the quality of evidence for each outcome as high, moderate, low, or very low [21]. The assessment included judgments addressing the risk of bias [22], imprecision [23], inconsistency [24], indirectness [25], and publication bias [26]. If there were serious concerns in any of these domains, we rated down the quality of evidence. 

## 3. Results

### 3.1. Search Strategy and Selection Criteria

**Figure 1:**
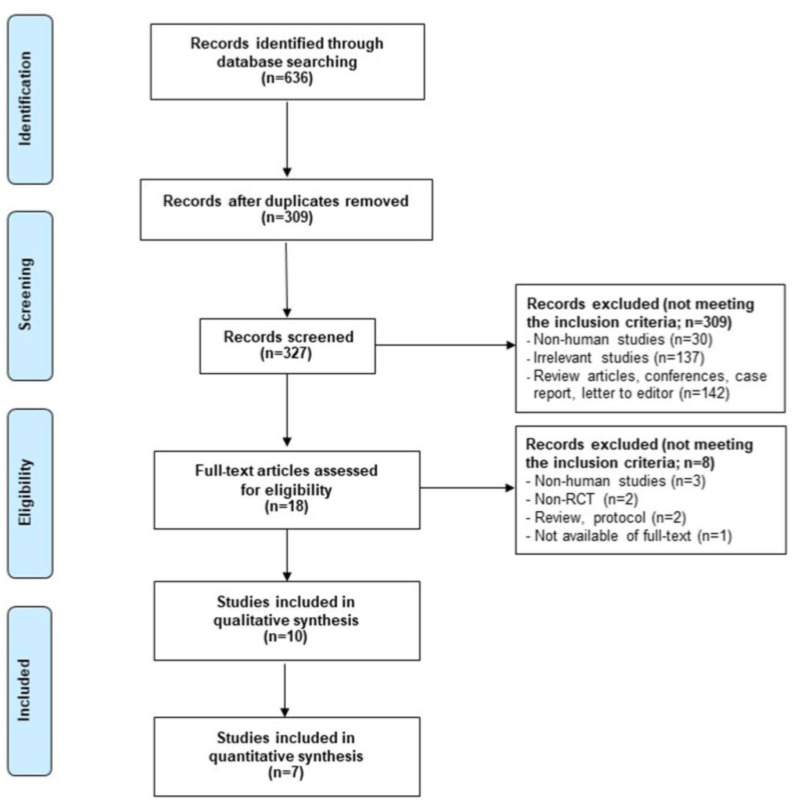
PRISMA flow diagram of study selection for the systematic review and meta-analysis.

In total, 636 citations were identified by the database search (Figure 1). After screening titles and abstracts, 18 full texts were screened. Eleven studies met our inclusion criteria, but the full text of Davey et al. [27] was not assessed. Finally, 10 studies [28,29,30,31,32,33,34,35,36,37] were included in our systematic review, 7 of which [29,30,31,32,33,35,36] were included in the meta-analysis. No additional articles were retrieved from the reference lists of the included studies.

### 3.2. Data Extraction and Risk-of-Bias Assessment

#### Study Characteristics

The study characteristics and the maternal and infant characteristics are shown in Table 1, and Supplementary Table S1, respectively. Among the 10 studies, 1 compared lactose hydrous (placebo) and intravenous (IV) hydrocortisone [28]. Three studies compared IV normal saline solution (NSS) or no treatment versus IV dexamethasone [29,35,36]. Two studies compared nebulized NSS, IV 5% dextrose, or no treatment (control) versus IV methylprednisolone or nebulized budesonide [30,31]. Two studies compared nebulized NSS versus nebulized budesonide [32,33]. One study assigned patients to receive either nebulized 3% sodium chloride with IV NSS (placebo) or nebulized budesonide with IV methylprednisolone [37]. One study [34], patients received intratracheal instillation of porcine lung surfactant (PS) or intratracheal instillation of PS with budesonide. Definitions used in the included studies were as follows:

1. MAS [28,29,30,31,32,33,34,36,37]
  - Delivery of MSAF infants and retrieval of meconium from below the larynx on endotracheal tube suction;
  - Development of respiratory distress within 4 to 6 h after birth and persistence beyond 24 h;
  - Chest X-ray findings of infiltrates, hyperinflation, and atelectasis;
  - Absence of any other causes of respiratory distress.
2. Sepsis [30]
  - Presence of clinical signs: poor feeding, weight loss, lethargy, temperature instability, sclerema, and capillary refill time of >3 s and;
  - Positive blood culture, or;
  - Two or more of the following laboratory abnormalities:
    (a) Total leukocyte count of <5000/mm^3^ or >30,000/mm^3^;
    (b) Immature/total neutrophil ratio of >0.2;
    (c) Micro-erythrocyte sedimentation rate of >5 mm in the first hour on the first day of life or >15 mm at any time;
    (d) Positive C-reactive protein.

### 3.3. Risk-of-Bias Assessment

The risk-of-bias assessment results are summarized in Supplementary Table S2. The allocation sequence was generated in 5 of 10 the studies [28,30,31,32,33], 1 of which used concealed allocation [30]. Three studies were double-blind RCTs [28,29,31] Loss to follow-up [28,29,30,31,32,33,34,35,36,37] and selective outcome reporting were adequate.

### 3.4. Data Analyses 

**Figure 2:**
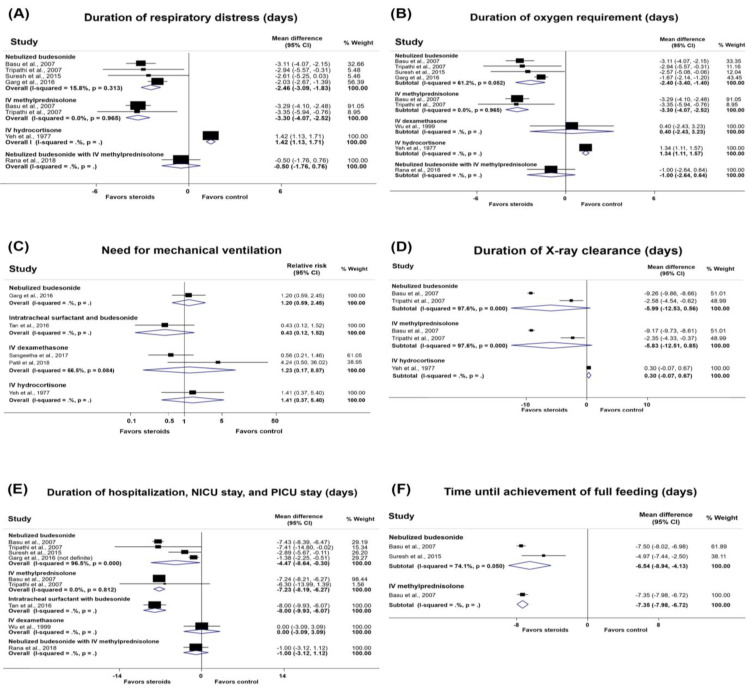
Forest plots of the effects of steroids in infants with meconium aspiration syndrome. (**A**) Duration of respiratory distress; (**B**) duration of oxygen requirement; (**C**) need for mechanical ventilation; (**D**) duration of X-ray clearance; (**E**) duration of hospitalization, NICU stay, and PICU stay; (**F**) time until achievement of full feeding.

**Figure 3:**
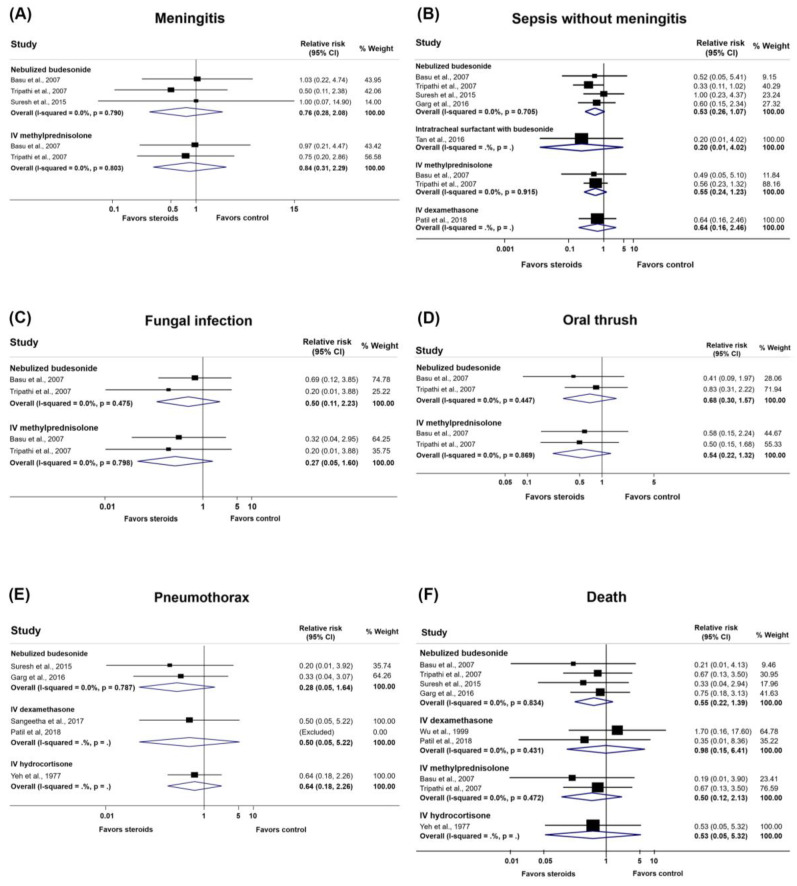
Forest plots of the complications and adverse effects of steroids in infants with meconium aspiration syndrome. (**A**) Meningitis; (**B**) sepsis without meningitis; (**C**) fungal infection; (**D**) oral thrush; (**E**) pneumothorax; (**F**) death.

The efficacy of steroids on clinical outcomes, ADRs, and complications in infants with MAS are shown in Figure 2 and Figure 3, Table 2, and Supplementary Tables S3, S4 and Figure S1.

#### 3.4.1. Duration of Respiratory Distress

Six RCTs provided evidence regarding respiratory distress [28,30,31,32,33,37]. With moderate-quality evidence, nebulized budesonide appeared to reduce the duration of respiratory distress in 4 RCTs [30,31,32,33] (MD, −2.46 days; 95% CI, −3.09 to −1.83 days) as well as IV methylprednisolone in 2 RCTs [30,31] (MD, −3.30 days; 95% CI, −4.07 to −2.52 days) (Figure 2A, Table 2, and Supplementary Tables S3 and S4). 

#### 3.4.2. Duration of Oxygen Requirement

Eight RCTs [28,29,30,31,32,33,36,37] provided data regarding the duration of oxygen requirement. Both nebulized budesonide and IV methylprednisolone appeared to reduce the duration of oxygen requirement. Four RCTs [30,31,32,33] demonstrated this beneficial effect of nebulized budesonide with low-quality evidence (MD, −2.4 days; 95% CI, −3.4 to −1.4 days). Two RCTs [30,31] demonstrated this beneficial effect of IV methylprednisolone with moderate-quality evidence (MD, −3.30 days; 95% CI, −4.07 to −2.52 days). Two RCTs [29,36] demonstrated the effect of dexamethasone, only one reported on the duration [29]. (Figure 2B, Table 2, and Supplementary Tables S3 and S4).

#### 3.4.3. Need for Mechanical Ventilation

Six RCTs [28,30,33,34,35,36] determined the effect of steroids on the need for mechanical ventilation. Two studies [35,36] showed no significant difference in the need for mechanical ventilation when using IV dexamethasone versus the control with very low-quality evidence (RR, 1.23; 95% CI, 0.17 to 8.87) (Figure 2C, Table 2, and Supplementary Tables S3 and S4).

#### 3.4.4. Duration of Mechanical Ventilation

Three RCTs [29,32,37] determined the effect of steroids on the duration of mechanical ventilation. Two RCTs [29,37] calculated the MD, which one RCT showed no difference in the duration of mechanical ventilation between dexamethasone and placebo (MD, −1.10 days; 95% CI, −2.79 to 0.59 days) [29].

#### 3.4.5. Downes’ Score

Two RCTs reported Downes’ score [33,37]. One study [33] reported a lower mean Downes’ score (over 5 days) in the nebulized budesonide group than in the control group (*p* < 0.05). The other study [37] showed a lower median Downes’ score (days 2–7) in the nebulized budesonide with IV methylprednisolone group than in the control group (*p* < 0.05).

#### 3.4.6. Duration of X-ray Clearance

Three RCTs [28,30,31] provided evidence regarding the duration of X-ray clearance. Two RCTs [30,31] determined this effect of nebulized budesonide and IV methylprednisolone with very low-quality evidence (MD, −5.99 days; 95% CI, −12.53 to 0.56 days and MD, −5.83 days; 95% CI, −12.51 to 0.85 days, respectively) (Figure 2D, Table 2, and Supplementary Tables S3 and S4). 

#### 3.4.7. Duration of Hospitalization, Neonatal Intensive Care Unit Stay, and Pediatric Intensive Care Unit Stay

Nine RCTs [29,30,31,32,33,34,35,36,37] provided evidence regarding the duration of hospitalization, including intensive care unit (ICU) admission. Four RCTs [30,31,32,33] showed that nebulized budesonide may shorten the duration of hospitalization with low-quality evidence (MD, −4.47 days; 95% CI, −8.64 to −0.30 days). Two RCTs [30,31] showed that IV methylprednisolone probably decreases the length of hospitalization with moderate-quality evidence (MD, −7.23 days; 95% CI, −8.19 to −6.07 days). Three RCTs [29,35,36] provided data on dexamethasone; among these, only one RCT [29] reported the duration of hospitalization (MD, 0.00 days; 95% CI, −3.09 to 3.09 days) (Figure 2E, Table 2, and Supplementary Tables S3 and S4)

#### 3.4.8. Time until Achievement of Full Feeding

Three RCTs [30,31,32] provided evidence regarding nebulized budesonide on time taken to full feeding in neonates with MAS. Two RCTs [30,32] showed a significantly shorter time until full feeding in the nebulized budesonide group than in the placebo or no treatment group with very low-quality evidence (MD, −6.54 days; 95% CI, −8.94 to −4.13 days) (Figure 2F and Supplementary Tables S3 and S4). 

#### 3.4.9. Duration of IV Fluid Requirement

One study showed that both nebulized budesonide and IV methylprednisolone reduced the duration of IV fluid requirement in infants with MAS [30] (MD, −6.95 days; 95% CI, −7.50 to −6.40 days and MD, −7.06 days; 95% CI, −7.66 to −6.46 days, respectively) (Supplementary Table S4 and Figure S1).

#### 3.4.10. Infections and Complications

We performed meta-analyses of infections and other complications, including pneumothorax, hypotension, hypoglycemia, hyperbilirubinemia, and seizure (Figure 3, Table 2, and Supplementary Tables S3, S4 and Figure S1). There was no significant association of steroids with infections and complications with low- to very low-quality evidence. A meta-analysis could not be performed for hypocalcemia, respiratory arrest, PPHN including the need for pulmonary vasodilators, anemia, stage 2 hypoxic-ischemic encephalopathy, and diarrhea (Supplementary Table S4 and Figure S1). One study [30] revealed no cases of hypertension or hyperglycemia in both the nebulized budesonide and IV methylprednisolone groups. One patient each in the nebulized budesonide and placebo groups developed hyperglycemia in one study [32].

#### 3.4.11. Death

Seven RCTs [28,29,30,31,32,33,36] provided data regarding death. A meta-analysis could be performed for six of these RCTs [29,30,31,32,33,36]. With low-quality evidence, the studies showed that mortality was not reduced with the use of IV dexamethasone [29,36] [RR, 0.98; 95% CI, 0.15 to 6.41; risk difference (RD), −0.23; 95% CI, −9.72 to 61.84], nebulized budesonide [30,31,32,33] (RR, 0.55; 95% CI, 0.22 to 1.39; RD, −5.14; 95% CI, −8.92 to 4.46), and IV methylprednisolone [30,31] (RR, 0.50; 95% CI, 0.12 to 2.13; RD, −5.72; 95% CI, −10.06 to 12.92).

## 4. Discussion

We included all available RCTs to evaluate the effectiveness, safety, and adverse effects of different types of steroids in infants with MAS. Our results show the benefits of both nebulized budesonide and IV methylprednisolone on the duration of respiratory distress, oxygen requirement, and hospitalization, including ICU admission (moderate- to low-quality evidence). Nebulized budesonide shortens the time until achievement of full feeding (very low-quality evidence) without statistically significant increases in the incidence of infections and complications (low- to very low-quality evidence). There was no reduction of mortality regardless of the type of steroid administration.

We performed a rigorous and systematic search to identify relevant studies using the revised version of the Cochrane risk-of-bias tool. We assessed the certainty of evidence for each outcome using the GRADE approach [16,21] our protocol was registered in PROSPERO, and reporting the results followed the PRISMA statement.

Based on the pathophysiology of MAS, treatments to reduce inflammation and cytokine production should benefit patients with MAS. The instillation of budesonide with surfactant has been shown to improve the respiratory status in animal studies [38]. Management of MAS in infants mainly involves supportive respiratory and cardiovascular care, with other modalities such as surfactants [39]. Insufficient treatment data in previous studies (e.g., steroids) were investigated in our study.

The effect of steroids on MAS in infants was evaluated in a Cochrane meta-analysis [15]; however, it included only studies by Yeh et al. [28] and Wu et al. [29] This meta-analysis showed no effect of steroids on the duration of oxygen therapy or mortality rate because of insufficient evidence.

Our study updated the current data regarding the effects of steroids on MAS with more relevant clinical outcomes and complications. In addition, other outcomes, such as pulmonary hypertension and respiratory arrest, were additionally identified and are shown in Supplementary Figure S1. 

There was no significant difference in the occurrence of PPHN among neonates with MAS with/without steroids [30,33,34,36]. Pneumothorax also showed no difference in neonates with/without budesonide [32,33]. No significant increase in either hypertension or hyperglycemia among neonates with steroids was reported [30,32].

### Strengths and Limitations

Our comprehensive and systematic search with separate and independent screening, searching, study selection, data extraction, quality assessment of this review focused on important outcomes. The GRADE approach was used to rate the quality of evidence, including risk of bias, inconsistency, indirectness, imprecision, and publication bias. 

There are several limitations. Even though only RCTs were evaluated, the quality varied from moderate to very low. For outcomes, the quality rating of evidence was decreased by one level based on the risk of bias (Table 2 and Supplementary Table S3). Adequate allocation sequence and concealment were reported in five studies [28,30,31,32,33] and one study [30], respectively. A blinding process was performed in three studies (Supplementary Table S2) [28,29,31]. For some outcomes, we decreased the quality of evidence rating by one level based on high heterogeneity (I^2^ > 50%) (Table 2 and Supplementary Table S3).

Notably, most of the included studies were performed in Asian populations, and 7 of the 10 studies were conducted in India or countries with low resource settings.

Patients were not severe as not much initial requirement of mechanical ventilation in most studies, and data on severity of pulmonary disease, such as oxygen index, were not provided. The analysis is also limited by the different methodology performed in each study and the relatively small number of infants available to assess each outcome. There is variability between the reported studies. Otherwise, the long-term outcomes of steroids, such as neurodevelopmental results, should be followed. Thus, the large-sample, uniform methodology and high-quality RCTs involving different populations should further confirm the effect of steroids in infants with MAS.

## 5. Conclusions

For infants with MAS, certain types of steroids may be beneficial in reducing the duration of respiratory distress, oxygen requirement, hospital stay, and time until achievement of full feeding without short-term complications. However, no benefits of decreased mortality in any types of steroid use.

## Figures and Tables

**Table 1:** Characteristics of included studies.

Study	Yeh et al., 1977 [28]	Wu et al., 1999 [29]	Basu et al., 2007 [30]	Tripathi et al., 2007 [31]	Suresh et al., 2015 [32]	Garg et al., 2016 [33]	Tan et al., 2016 [34]	Sangeetha et al., 2017 [35]	Patil et al., 2018 [36]	Rana et al., 2018 [37]
Type of study	RCT	RCT	RCT	RCT	RCT	RCT	RCT	RCT	RCT	RCT
Location	Illinois, USA	Taipei, Taiwan	Varanasi, India	New Delhi, India	Karnataka, India	Rajasthan, India	Foshan Nanhai, China	Chidambaram, India	Karnataka, India	West Bengal, India
Inclusion criteria	- Neonates with MAS	- Neonates with MAS	- Neonates with MAS	- Full-term neonates - BW > 2000 g - MAS	- Full-term neonates - MAS	- Term neonates - (GA ≥ 37 weeks) - BW ≥ 2000 g - Neonates with non-vigorous meconium-stained amniotic fluid - Neonates with MAS	- Neonates with MAS	- Term neonates with MAS - Admitted to NICU	- GA ≥ 34 weeks - Neonates with MAS	- Neonates with MAS
Exclusion criteria	-	-	- Sepsis - Systemic illness - Gross congenital malformations	- Preterm - IUGR - Out-born babies - Congenital malformation - Denied consent	- Sepsis - Systemic illness - Gross congenital malformation - Preterm - IUGR - Denied consent	- Congenital malformation - Grade 3 HIE - History suggestive of early-onset sepsis	- Severe congenital malformations - Serious systemic diseases - Heart failure - Renal insufficiency - Acute or chronic diseases	- Major congenital anomalies - Congenital heart disease	- Congenital malformation - Denied consent - Suspected sepsis	- Preterm - IUGR - Major congenital malformations - Denied consent - Died during early neonatal period
Randomization	Random number table	Randomization method not mentioned	Computer-generated random numbers	Computer-generated random numbers	Computer-generated random numbers	Computer-generated random numbers	Randomization method not mentioned	Randomization method not mentioned	Randomization method not mentioned	Randomization method not mentioned
Study period	1 year (October 1974 to September 1975)	NR	1 year	1 year	3 months (August to October 2013)	5 months (May 2010 to December 2010)	2 years (December 2013 to December 2015)	1 year	18 months	3 years (April 2014 to March 2017)
All groups received	Standard care as protocol	Standard care as protocol	Supportive treatment as protocol of nursery	Standard care as unit protocol	Supportive treatment as protocol of nursery	Standard care as protocol	PS 100 mg/kg within 2 h	Standard care as unit protocol	Standard care as protocol	Standard care
Control	Placebo (lactose hydrous)	NSS	None	- Nebulized NSS - 5% Dextrose IV	Nebulized NSS	- Nebulized NSS 2.5 mL within 2 h and at 12 h after birth via nebulizer system	- PS 100 mg/kg by intratracheal instillation within 2 h of birth	None	None	- Nebulized 3% NaCl - IV NSS
Comparator (1)	Hydrocortisone 20 mg/kg IV for first bolus dose, then continue q 12 h for 4 more doses	Dexamethas1 mg/kg IV for initial dose, then 0.5 mg/kg q 12 h for days 1–3, then 0.25 mg/kg q 12 h for days 4–7 (started shortly after birth)	Methylprednisolone 0.5 mg/kg/day IV q 12 h for 7 days (started 24–36 h after birth)	- Nebulized NSS - Methylprednisolone 0.5 mg/kg/day IV q 12 h for 7 days	- Nebulized budesonide 50 µg with NSS 2.5 mL via jet nebulizer q 12 h for 7 days or clinical recovery (whichever occurred first) (started day 2 after birth)	- Nebulized budesonide 0.5 mg with NSS 2.5 mL within 2 h of birth and second dose at 12 h after birth (= budesonide 50 µg) via nebulizer system	- PS 100 mg/kg with budesonide 0.25 mg/kg by intratracheal instillation within 2 h of admission	Dexamethasone 0.5 mg/kg IV q 12 h for days 1–3, then 0.25 mg/kg q 12 h for days 4–7	- Dexamethasone 0.25 mg/kg IV q 12 h for 3 days (started 24–36 h after birth)	- Methylprednisolone 0.5 mg/kg/day q 12 h - Nebulized budesonide respirator suspension 0.5 mg + NSS 2.5 mL q 12 h for 7 days
Comparator (2)			Nebulized budesonide 50 µg + NSS 2.5 mL via jet nebulizer q 12 h for 7 days (started 24–36 h after birth)	- Nebulized budesonide 50 µg + NSS 2.5 mL via jet nebulizer q 12 h for 7 days - 5% Dextrose IV	-	-	-	-		-
Follow up	-	-	Weekly for 2 weeks, then monthly	-	Once every 2 weeks for 3 months	-	-	-	-	1, 3, and 6 months for any complications
Outcomes	Need for mechanical ventilation Air leakage Mortality A-a oxygen gradient Duration of oxygen dependence Duration of respiratory distress	Duration of mechanical ventilation Mortality Chronic lung disease morbidities Duration of oxygen dependence Duration of hospital stay Transient elevation of blood pressure and glucose Weight loss Pulmonary pressure Acid-base status	Duration of respiratory distress Oxygen requirement Duration of hospital stay Requirement of IV fluids Starting feeding Achievement of full feeding CXR clearance	Efficacy outcomes: Duration of stay Survival Duration of oxygen dependence Duration of X-ray clearance Feeding Discontinuation of IV fluids Safety outcomes: - Infection rates - Hypertension - Hypotension requiring vasopressor - Need for blood products - Hyperbilirubinemia - Hypoglycemia - Seizures	Duration of respiratory distress Duration of oxygen dependency Duration of hospital stay Time until full feeding Need for mechanical ventilation Complications: - Meningitis - Sepsis without meningitis - Hypotension - Pneumothorax - Seizures - Hyperglycemia - Hypoglycemia - Hypocalcemia - Hyperbilirubinemia - Mortality	Respiratory score (Downes’ score) Requirement (dependence) of oxygen Duration of NICU stay Complications	Repeated use of PS Need for mechanical ventilation Improvement of PaO_2_/FiO_2_, TcSaO_2_, PaO_2_, and PCO_2_ CXR improvement after 48 h Duration of hospitalization Complications	Duration of hospital stay Air-leak syndrome (pneumothorax) Need for ventilation support	Duration of oxygen dependency Duration of hospital stay Initiation of oral feeding Sepsis Need for mechanical ventilation Pulmonary vasodilator needed Stage 2 or 3 HIE Air-leak syndrome Death	Respiratory score (Downes’ score) Requirement (dependence) of oxygen Time until resolution of respiratory distress Days on mechanical ventilation Duration of hospitalization Long-term complications

**Abbreviations:** A-a oxygen gradient = alveolar-arterial oxygen gradient; BW = birth weight; CXR = chest X-ray; FiO2 = fraction of inspired oxygen; GA = gestational age; HIE = hypoxic-ischemic encephalopathy; IUGR = intrauterine growth restriction; IV = intravenous; MAS = meconium aspiration syndrome; NaCl = sodium chloride; NICU = neonatal intensive care unit; NR = not reported; NSS = normal saline solution; PaO_2_ = partial pressure of arterial oxygen; PaCO_2_ = partial pressure of arterial carbon dioxide; PS = porcine lung surfactant; RCT = randomized controlled trial; TcSaO_2_ = transcutaneous arterial oxygen saturation.

**Table 2:** GRADE summary of findings: Effect of steroids on clinical outcomes in neonates with MAS.

**Patient or Population:** Neonates with MAS **Intervention:** Steroids **Comparison:** Placebo, no treatment, or usual care						
**Outcomes**	**No. of Participants** **(Studies)**	**Relative Effects** **(95% CI)**	**Absolute Effect Estimates**		**Certainty/Quality of Evidence**	**Plain Language Summary**
			**Baseline Risk for Control Group ^1^**	**Difference (95% CI)**		
**Duration of respiratory distress (days)**						
Budesonide	208 (4 studies)	-	The median duration of respiratory distress in the control groups was 5.71 days	MD −2.46 days (−3.09 to −1.83)	Moderate ⊕⊕⊕⊝ (serious risk of bias)	Budesonide probably reduces duration of respiratory distress.
Methylprednisolone	96 (2 studies)	-	The median duration of respiratory distress in the control groups was 5.71 days	MD −3.30 days (−4.07 to −2.52)	Moderate ⊕⊕⊕⊝ (serious risk of bias)	Methylprednisolone probably reduces duration of respiratory distress.
**Duration of oxygen requirement (days)**						
Budesonide	208 (4 studies)	-	The median duration of oxygen requirement in the control groups was 4.94 days	MD −2.40 days (−3.40 to −1.40)	Low ⊕⊕⊝⊝ (serious risk of bias, serious inconsistency)	Budesonide may reduce duration of oxygen requirement.
Methylprednisolone	96 (2 studies)	-	The median duration of oxygen requirement in the control groups was 4.94 days	MD −3.30 days (−4.07 to −2.52)	Moderate ⊕⊕⊕⊝ (serious risk of bias)	Methylprednisolone probably reduces duration of oxygen requirement.
**Need for mechanical ventilation**						
Dexamethasone	130 (2 studies)	1.23 (0.17 to 8.87)	25.00%	5.75 (−20.75 to 196.75)	Very low ⊕⊝⊝⊝ (serious risk of bias, serious inconsistency, and serious imprecision)	The effect of dexamethasone on the need for mechanical ventilation is very uncertain.
**Duration of hospitalization, NICU stay, and PICU stay (days)**						
Budesonide	208 (4 studies)	-	The median duration of hospitalization/NICU stay in the control groups was 14 days	MD −4.47 days (−8.64 to −0.30)	Low ⊕⊕⊝⊝ (serious risk of bias, serious inconsistency)	Budesonide may reduce the duration of hospitalization, NICU stay, and PICU stay.
Methylprednisolone	96 (2 studies)	-	The median duration of hospitalization/NICU stay in the control groups was 14 days	MD −7.23 days (−8.19 to −6.27)	Moderate ⊕⊕⊕⊝ (serious risk of bias)	Methylprednisolone probably reduces the duration of hospitalization, NICU stay, and PICU stay.
***Infections and death***						
**Meningitis**						
Budesonide	139 (3 studies)	0.76 (0.28 to 2.08)	10.00%	−2.40 (−7.20 to 10.80)	Low ⊕⊕⊝⊝ (serious risk of bias, serious imprecision)	Budesonide may not increase the number of participants with meningitis.
Methylprednisolone	101 (2 studies)	0.84 (0.31 to 2.29)	10.00%	−1.60 (−6.90 to 12.90)	Low ⊕⊕⊝⊝ (serious risk of bias, serious imprecision)	Methylprednisolone may not increase the number of participants with meningitis.
**Sepsis without meningitis**						
Budesonide	217 (4 studies)	0.53 (0.26 to 1.07)	15.42%	−7.25 (−11.41 to 1.08)	Low ⊕⊕⊝⊝ (serious risk of bias, serious imprecision)	Budesonide may not increase the number of participants with sepsis without meningitis.
Methylprednisolone	101 (2 studies)	0.55 (0.24 to 1.23)	15.42%	−6.94 (−11.72 to 3.55)	Low ⊕⊕⊝⊝ (serious risk of bias, serious imprecision)	Methylprednisolone may not increase the number of participants with sepsis without meningitis.
**Death**						
Budesonide	217 (4 studies)	0.55 (0.22 to 1.39)	11.43%	−5.14 (−8.92 to 4.46)	Low ⊕⊕⊝⊝ (serious risk of bias, serious imprecision)	Budesonide may not increase the number of participants with death.
Dexamethasone	120 (2 studies)	0.98 (0.15 to 6.41)	11.43%	−0.23 (−9.72 to 61.84)	Low ⊕⊕⊝⊝ (serious risk of bias, serious imprecision)	Dexamethasone may not increase the number of participants with death.
Methylprednisolone	101 (2 studies)	0.50 (0.12 to 2.13)	11.43%	−5.72 (−10.06 to 12.92)	Low ⊕⊕⊝⊝ (serious risk of bias, serious imprecision)	Methylprednisolone may not increase the number of participants with death.

**Abbreviations:** CI = confidence interval; RCTs = randomized controlled trials; MAS = meconium aspiration syndrome; MD = mean difference; NICU = neonatal intensive care unit; PICU = pediatric intensive care unit. **Footnote:** ^1^ Using the median baseline risk in the control group of eligible RCTs. GRADE Working Group grades of evidence. High certainty: We are very confident that the true effect lies close to that of the estimate of the effect. Moderate certainty ⊕⊕⊕⊝: We are moderately confident in the effect estimate: the true effect is likely to be close to the estimate of the effect, but there is a possibility that it is substantially different. Low certainty ⊕⊕⊝⊝: Our confidence in the effect estimate is limited: the true effect may be substantially different from the estimate of the effect. Very low certainty ⊕⊝⊝⊝: We have very little confidence in the effect estimate: the true effect is likely to be substantially different from the estimate of the effect.

## Data Availability

The data supporting the findings of this study are available within the article.

## Supplementary Materials

The following are available online at https://www.mdpi.com/article/10.3390/medicina57111281/s1. Supplement Table S1: baseline maternal and neonatal characteristics of the included studies; Supplement Table S2: risk-of-bias summary of the included studies using revised Cochrane risk-of-bias tool for randomized trials; Supplement Table S3: GRADE evidence profile of the evidence outcomes; Supplement Table S4: summary results of the included studies categorized by outcomes; Supplement Figure S1: results of the outcomes in the systematic review and meta-analysis.
