# Supplementary material for: The Effects of Different Types of Steroids on Clinical Outcomes in Neonates with Meconium Aspiration Syndrome: A Systematic Review, Meta-Analysis and GRADE Assessment
Source: Medicina (Kaunas). 2021 Nov 21;57(11):1281. doi: 10.3390/medicina57111281 (PMC8619455; doi:10.3390/medicina57111281)
Supplement: Supplementary file 1 [file medicina-57-01281-s001.zip › medicina-1439305-supplementary.pdf]

Supplementary Materials

Supplementary Table S1. Baseline maternal and neonatal characteristics of the included studies

Supplementary Table S2. Risk-of-bias summary of the included studies using Revised Cochrane risk-of-bias tool for randomized trials

Supplementary Table S3. GRADE evidence profile of the evidence outcomes

Supplementary Table S4. Summary results of the included studies categorized by outcomes

Supplementary Figure S1. Results of the outcomes in the systematic review and meta-analysis

Supplementary Table S1. Baseline characteristics of maternal and neonatal in the included studies

Study	Steroids		Control
**Yeh et al., 1977 [28]**	**IV hydrocortisone (n = 17)**		**IV lactose hydrous (n = 18)**
Maternal age (years), mean±SD	NR		NR
ANC, n (%)	NR		NR
Non-NSVD, n (%)	NR		NR
Gestational age (weeks), mean±SD	40.7±0.4		40.8±0.3
Birth weight (g), mean±SD	3100±130		3200±120
Apgar score at 1 min, mean±SD	5 ±0.6		4.2±0.6
Apgar score at 5 min, mean±SD	5.7±0.8		5.8±0.7
RDS at admission, mean±SD	4.6±0.4		4.3±0.4
FiO_2 on admission, mean±SD	0.61±0.06		0.56±0.04
**Wu et al., 1999 [29]**	**IV dexamethasone (n = 27)**		**IV NSS (n = 23)**
Maternal age (years), mean±SD	NR		NR
ANC, n (%)	NR		NR
Non-NSVD, n (%)	NR		NR
Gestational age (weeks), mean±SD	NR		NR
Birth weight (g), mean±SD	NR		NR
Male, n (%)	NR		NR
FiO_2 on admission, mean±SD	0.52±0.24		0.54±0.31
**Basu et al., 2007 [30]**	**Nebulized budesonide (n = 32)**	**IV methylprednisolone (n = 34)**	**None (n = 33)**
Maternal age (years), mean±SD	25.0±5.3	25.7±4.8	24.4±4.7
Gravida, mean±SD	2.9±1.5	2.9±1.5	2.9±1.4
Non-NSVD (instrument and C-section), n (%)	25 (78)	25(74)	27 (82)
Thick meconium, n (%)	17 (53)	19 (56)	20 (61)
Gestational age (weeks), mean±SD	38.9±2.9	39.0±2.8	38.6±2.8
Birth weight (g), mean±SD	2610±500	2780±1680	2580±490
Male, n (%)	18 (56)	21 (62)	19 (58)
Apgar score at 1 min, mean±SD	4.2±1.6	4.8±1.7	5.0±2.2
Apgar score at 5 min, mean±SD	5.8±1.4	5.7±1.4	5.8±1.4
Apgar score at 10 min, mean±SD	6.9±1.2	6.9±1.3	7.0±1.3
Intratracheal suctioning, n (%)	32 (100)	34 (100)	33 (100)
RDS at admission, mean±SD	3.1±1.9	3.2±1.1	3.4±1.3
**Tripathi et al., 2007 [31]**	**Nebulized budesonide (n = 17)**	**IV methylprednisolone (n = 17)**	**Nebulized NSS and**

			**IV 5% dextrose (n = 17)**
Maternal age (years), mean±SD	NR	NR	NR
ANC, n (%)	NR	NR	NR
Non-NSVD (instrument and C-section), n (%)	10 (59)	9 (53)	11 (65)
Birth weight (g), mean±SD	2800±370	2630±450	2640±340
Apgar score at 1 min, mean±SD	2.3±2.0	3.7±2.1	4.3±2.3
Apgar score at 5 min, mean±SD	6.1±1.0	6.9±1.3	6.5±1.8
Apgar score at 10 min, mean±SD	7.4±1.4	7.9±1.1	7.7±1.2
Infants with HIE, n (%)	3 (18)	3 (18)	4 (24)
**Suresh et al., 2015 [32]**	**Nebulized budesonide (n = 20)**		**Nebulized NSS (n = 20)**
Maternal age (years), mean±SD	NR		NR
ANC, n (%)	NR		NR
Non-NSVD (instrument and C-section), n (%)	13 (65)		12 (60)
Birth weight (g), mean±SD	2820±167		2815±195
Male, n (%)	15 (75)		13 (65)
Apgar score at 5 min, mean±SD	6.0±0.8		6.2±0.9
RDS at initiation of treatment, mean±SD	3.8±0.8		4.0±0.7
**Garg et al., 2016 [33]**	**Nebulized budesonide (n = 39)**		**Nebulized NSS (n = 39)**
Maternal age (years), mean±SD	NR		NR
ANC, n (%)	NR		NR
Non-NSVD (instrument and C-section), n (%)	NR		NR
Male, n (%)	22 (58)		22 (58)
**Tan et al., 2016 [34]**	**Intratracheal PS with budesonide (n = 35)**		**Intratracheal PS (n = 35)**
Maternal age (years), mean±SD	NR		NR
ANC, n (%)	NR		NR
Non-NSVD (instrument and C-section), n (%)	NR		NR
Gestational age (weeks), mean±SD	40±3		39±3
Birth weight (g), mean±SD	3437±594		3442±524
Male, n (%)	21 (60)		20 (57)
Age at onset of respiratory distress, mean (hours), mean±SD	8±4		8±3
**Sangeetha et al., 2017 [35]**	**IV dexamethasone (n = 30)**		**None (n = 30)**
Maternal age (years), mean±SD	NR		NR
ANC, n (%)	NR		NR
Non-NSVD (instrument and C-section), n (%)	NR		NR

Gestational age (weeks), mean±SD	NR	NR
Birth weight (g), mean±SD	NR	NR
Male, n (%)	NR	NR
**Patil et al., 2018 [36]**	**IV dexamethasone (n = 34)**	**None (n = 36)**
Maternal age (years), mean±SD	25.3±2.3	24.9±2.0
ANC, n (%)	26 (77)	27 (75)
Non-NSVD (instrument and C-section), n (%)	8 (24)	10 (28)
Primigravida, n (%)	22 (65)	20 (56)
Gestational age (weeks), mean±SD	38.90±1.2	38.8±1.1
Birth weight (g), mean±SD	2700±300	2700±400
Male, n (%)	20 (59)	21 (58)
Apgar score at 1 min, mean±SD	8.5±1.0	8.3±1.3
Apgar score at 5 min, mean±SD	8.5±0.5	8.7±0.5
Downes' score on admission, mean±SD	3.6±1.6	3.5±1.5
**Rana et al., 2018 [37]**	**Nebulized budesonide with IV methylprednisolone (n = 137)**	**Nebulized 3% NaCl with IV NSS (n = 138)**
Maternal age (years), mean±SD	NR	NR
ANC, n (%)	NR	NR
Non-NSVD (instrument and C-section), n (%)	66 (48)	66 (48)
Gestational age (weeks)<br>Term, n (%)<br>Post-term, n (%)	 98 (72)<br>39 (29)	 100 (73)<br>38 (28)
Birth weight > 2000 g, n (%)	137 (100)	138 (100)
Male, n (%)	70 (51)	71 (51)
Downes' score on day 1, mean±SD	3.00±3.70*	3.00±3.70*

Abbreviations: ANC = antenatal care; C-section = Cesarean section; HIE = hypoxic-ischemic encephalopathy; IV = intravenous; NaCl = sodium chloride; NSS = normal saline solution; NSVD = normal spontaneous vaginal delivery; NR = not reported; PS = porcine lung surfactant; RDS = respiratory distress score; SD = standard deviation
*Converted data from median (interquartile range) to mean (SD)

Supplementary Table S2. Risk-of-bias summary of the included studies using Revised Cochrane risk-of-bias tool for randomized trials[1]

Author (year)	Was the allocation sequence adequately generated?	Was the allocation adequately concealed?	Blinding: Was knowledge of the allocated interventions adequately prevented?					Was loss to follow-up (missing outcome data) infrequent?	Are reports of the study free of selective outcome reporting?	Was the study apparently free of other problems that could put it at a risk of bias?
			Were patients blinded?	Were healthcare providers blinded?	Were data collectors blinded?	Were outcome assessors blinded?	Were data analysts blinded?			
Yeh et al., 1977	Y	PN	Y	Y	N	Y	PN	Y	PY	PY
Wu et al., 1999	PN	PN	Y	Y	N	Y	PN	Y	PY	PY
Basu et al., 2007	Y	Y	PN	N	N	N	PN	Y	PN	PY
Tripathi et al., 2007	Y	PN	Y	Y	N	Y	PN	Y	PY	PY
Suresh et al., 2015	Y	PN	PN	N	PN	N	PN	Y	PY	PY
Garg et al., 2016	Y	PN	PN	PN	PN	PN	PN	Y	PY	PY
Tan et al., 2016	PN	PN	PN	PN	PN	PN	PN	Y	PY	PY
Sangeetha et al., 2017	PN	PN	PN	PN	PN	PN	PN	Y	PY	PY
Patil et al., 2018	PN	PN	PN	PN	PN	PN	PN	Y	PN	PY
Rana et al., 2018	PN	PN	PN	PN	PN	PN	PN	Y	PN	PY

Abbreviations: PN = probably no; PY = probably yes; N = definitely no; Y = definitely yes

Footnote: [1]Definitely yes and probably yes were defined as low risk of bias; definitely no and probably no were defined as high risk of bias.

Supplementary Table S3. GRADE evidence profile of the evidence outcomes

	Interventions	No. of participants (studies)	Quality assessment					Summary of findings			
			Risk of bias	Inconsistency	Indirectness	Imprecision	Publication Bias	Relative effects	Estimation of absolute effects		Quality of evidence
									Control risk or control median duration[1]	Mean difference or risk difference (95% CI)	
Efficacy of steroids	*Duration of respiratory distress (days)*										
	Budesonide	208 (4 studies)	Serious	Not serious	Not serious	Not serious	Undetected	-	The median duration of respiratory distress in the control groups was 5.17 days	MD –2.46 days (–3.09 to –1.83)	Moderate ⊕⊕⊕⊖ (Serious risk of bias)
	Methylprednisolone	96 (2 studies)	Serious	Not serious	Not serious	Not serious	Undetected	-	The median duration of respiratory distress in the control groups was 5.17 days	MD –3.30 days (–4.07 to –2.52)	Moderate ⊕⊕⊕⊖ (Serious risk of bias)
	Duration of oxygen requirement (days)										
	Budesonide	208 (4 studies)	Serious	Serious	Not serious	Not serious	Undetected	-	The median duration of oxygen requirement in the control groups was 4.94 days	MD –2.40 days (–3.40 to –1.40)	Low ⊕⊕⊖⊖ (Serious risk of bias, serious inconsistency)
	Methylprednisolone	96 (2 studies)	Serious	Not serious	Not serious	Not serious	Undetected	-	The median duration of oxygen requirement in the control groups was 4.94 days	MD –3.30 days (–4.07 to –2.52)	Moderate ⊕⊕⊕⊖ (Serious risk of bias)
	Need for mechanical ventilation										
	Dexamethasone	130 (2 studies)	Serious	Serious	Not serious	Serious	Undetected	1.23 (0.17–8.87)	25.00%	5.75 (–20.75 to 196.75)	Very low ⊕⊖⊖⊖ (Serious risk of bias, serious inconsistency, and serious imprecision)
	Duration of X-ray clearance (days)										

	Budesonide	94 (2 studies)	Serious	Serious	Not serious	Serious	Undetected	-	The median duration of X-ray clearance in the control groups was 7.76 days	MD −5.99 days (−12.53 to 0.56)	Very low ⊕⊖⊖⊖ (Serious risk of bias, serious inconsistency, and serious imprecision)
	Methylprednisolone	96 (2 studies)	Serious	Serious	Not serious	Serious	Undetected	-	The median duration of X-ray clearance in the control groups was 7.76 days	MD −5.83 days (−12.51 to 0.85)	Very low ⊕⊖⊖⊖ (Serious risk of bias, serious inconsistency, and serious imprecision)
	Duration of hospitalization, NICU stay, and PICU stay (days)										
	Budesonide	208 (4 studies)	Serious	Serious	Not serious	Not serious	Undetected	-	The median duration of hospitalization/NICU stay in the control groups was 14.00 days	MD −4.47 days (−8.64 to −0.30)	Low ⊕⊕⊖⊖ (Serious risk of bias, serious inconsistency)
	Methylprednisolone	96 (2 studies)	Serious	Not serious	Not serious	Not serious	Undetected	-	The median duration of hospitalization/NICU stay in the control groups was 14.00 days	MD −7.23 days (−8.19 to −6.27)	Moderate ⊕⊕⊕⊖ (Serious risk of bias)
	Time until achievement of full feeding (days)										
	Budesonide	101 (2 studies)	Serious	Serious	Serious	Not serious	Undetected	-	The median duration until achievement of full feeding in the control groups was 11.34 days	MD −6.54 days (−8.94 to −4.13)	Very low ⊕⊖⊖⊖ (Serious risk of bias, serious indirectness, and serious inconsistency)
**Infection and complications**	***Infections***										
	**Meningitis**										
	Budesonide	139 (3 studies)	Serious	Not serious	Not serious	Serious	Undetected	0.76 (0.28–2.08)	10.00%	−2.40 (−7.20 to 10.80)	Low ⊕⊕⊖⊖ (Serious risk of bias, serious imprecision)
	Methylprednisolone	101 (2 studies)	Serious	Not serious	Not serious	Serious	Undetected	0.84 (0.31–2.29)	10.00%	−1.60 (−6.90 to 12.90)	Low ⊕⊕⊖⊖ (Serious risk of bias, serious imprecision)
	**Sepsis without meningitis**										
	Budesonide	217 (4 studies)	Serious	Not serious	Not serious	Serious	Undetected	0.53 (0.26–1.07)	15.42%	−7.25 (−11.41 to 1.08)	Low ⊕⊕⊖⊖ (Serious risk of bias, serious imprecision)
	Methylprednisolone	101	Serious	Not serious	Not serious	Serious	Undetected	0.55 (0.24–1.23)	15.42%	−6.94 (−11.72 to 3.55)	Low ⊕⊕⊖⊖

		(2 studies)									(Serious risk of bias, serious imprecision)
	**Fungal infection**										
	Budesonide	99 (2 studies)	Serious	Not serious	Not serious	Serious	Undetected	0.50 (0.11–2.23)	11.67%	−5.84 (−10.39 to 14.35)	Low ⊕⊕⊖⊖ (Serious risk of bias, serious imprecision)
	Methylprednisolone	101 (2 studies)	Serious	Not serious	Not serious	Serious	Undetected	0.27 (0.05–1.60)	11.67%	−8.52 (−11.09 to 7.00)	Low ⊕⊕⊖⊖ (Serious risk of bias, serious imprecision)
	**Oral thrush**										
	Budesonide	99 (2 studies)	Serious	Not serious	Not serious	Serious	Undetected	0.68 (0.30–1.57)	36.20%	−11.58 (−25.34 to 20.63)	Low ⊕⊕⊖⊖ (Serious risk of bias, serious imprecision)
	Methylprednisolone	101 (2 studies)	Serious	Not serious	Not serious	Serious	Undetected	0.54 (0.22–1.32)	36.20%	−16.65 (−28.24 to 11.58)	Low ⊕⊕⊖⊖ (Serious risk of bias, serious imprecision)
	Other complications										
	**Pneumothorax**										
	Budesonide	118 (2 studies)	Serious	Not serious	Not serious	Serious	Undetected	0.28 (0.05–1.64)	9.72%	−7.00 (−9.23 to 6.22)	Low ⊕⊕⊖⊖ (Serious risk of bias, serious imprecision)
	**Hypotension**										
	Budesonide	105 (2 studies)	Serious	Not serious	Not serious	Serious	Undetected	0.85 (0.27–2.62)	13.82%	−2.07 (−10.09 to 22.39)	Low ⊕⊕⊖⊖ (Serious risk of bias, serious imprecision)
	**Hypoglycemia**										
	Budesonide	105 (2 studies)	Serious	Not serious	Serious	Serious	Undetected	0.92 (0.41–2.07)	22.29%	−1.78 (−13.15 to 23.85)	Very low ⊕⊖⊖⊖ (Serious risk of bias, serious indirectness, and serious imprecision)
	**Hyperbilirubinemia**										
	Budesonide	105 (2 studies)	Serious	Not serious	Serious	Serious	Undetected	1.02 (0.39–2.70)	17.86%	0.36 (−10.89 to 30.36)	Very low ⊕⊖⊖⊖ (Serious risk of bias, serious indirectness, and serious imprecision)

	**Seizure**										
	Budesonide	105 (2 studies)	Serious	Not serious	Serious	Serious	Undetected	0.31 (0.05–1.92)	10.56%	−7.29 (−10.03 to 9.72)	Very low ⊕⊖⊖⊖ (Serious risk of bias, serious indirectness, and serious imprecision)
	**Death**										
	Budesonide	217 (4 studies)	Serious	Not serious	Not serious	Serious	Undetected	0.55 (0.22–1.39)	11.43%	−5.14 (−8.92 to 4.46)	Low ⊕⊕⊖⊖ (Serious risk of bias, serious imprecision)
	Dexamethasone	120 (2 studies)	Serious	Not serious	Not serious	Serious	Undetected	0.98 (0.15–6.41)	11.43%	−0.23 (−9.72 to 61.84)	Low ⊕⊕⊖⊖ (Serious risk of bias, serious imprecision)
	Methylprednisolone	101 (2 studies)	Serious	Not serious	Not serious	Serious	Undetected	0.50 (0.12–2.13)	11.43%	−5.72 (−10.06 to 12.92)	Low ⊕⊕⊖⊖ (Serious risk of bias, serious imprecision)

Abbreviations: CI = confidence interval; MD = mean difference; NICU = neonatal intensive care unit; PICU = pediatric intensive care unit

Footnote:

1 Median baseline risk was used for the control group in the eligible randomized controlled trials.

Supplementary Table S4. Summary results of the included studies categorized by outcomes

	Authors (year)	Intervention	Steroids	Control	Mean difference (95% CI)
			Mean±SD	Mean±SD	
**Outcomes**	**Duration of respiratory distress (day)**				
	Yeh et al., 1977	IV hydrocortisone	3.32±0.48	1.9±0.37	1.42 (1.1, 1.71)
	Overall (I^2 = %, P =)				1.42 (1.13, 1.71)
	Basu et al., 2007	Nebulized budesonide	4.59±2.26	7.70±1.6	−3.11 (−4.07, −2.15)
	Tripathi et al., 2007	Nebulized budesonide	4.06±1.52	7.00±4.81	−2.94 (−5.57, −0.31)
	Suresh et al., 2015	Nebulized budesonide	2.63±0.96	5.24±5.48	−2.61 (−5.25, 0.03)
	Garg et al., 2016	Nebulized budesonide	3.06±1.43	5.09±1.46	−2.03 (−2.67, −1.39)
	Overall (I^2 = 15.8%, P =0.313)				−2.46 (−3.09, −1.83)
	Basu et al., 2007	IV methylprednisolone	4.41±1.76	7.70±1.63	−3.29 (−4.10, −2.48)
	Tripathi et al., 2007	IV methylprednisolone	3.65±1.18	7.00±4.81	−3.35 (−5.94, −0.76)
	Overall (I^2= 0.0%, P = 0.965)				−3.30 (−4.07, −2.52)
	Rana et al., 2018	Nebulized budesonide and IV methylprednisolone	3.00±1.48*	3.50±7.4*	−0.50 (−1.76, 0.76)
	Overall (I^2 = %, P =)				−0.50 (−1.76, 0.76)
	**Duration of oxygen requirement (days)**				
	Yeh et al., 1977	IV hydrocortisone	2.87±0.4	1.53±0.29	1.34 (1.11, 1.57)
	Overall (I^2 = %, P =)				1.34 (1.11, 1.57)
	Wu et al., 1999	IV dexamethasone	7.00± 4.10	6.60±5.80	0.40 (−2.43, 3.23)
	Overall (I^2 = %, P =)				0.40 (−2.43, 3.23)
	Basu et al., 2007	Nebulized budesonide	4.59±2.26	7.70±1.63	−3.11 (−4.07, −2.15)
	Tripathi et al., 2007	Nebulized budesonide	4.06±1.52	7.00±4.81	−2.94 (−5.57, −0.31)
	Suresh et al., 2015	Nebulized budesonide	2.37±0.60	4.94±5.24	−2.57 (−5.08, −0.06)
	Garg et al., 2016	Nebulized budesonide	1.79±0.95	3.46±1.15	−1.67 (−2.14, −1.20)
	Overall (I^2 = 61.2%, P =0.052)				−2.40 (−3.40, −1.40
	Basu et al., 2007	IV methylprednisolone	4.41±1.76	7.70±1.63	−3.29 (−4.10, −2.48)

	Tripathi et al., 2007	IV methylprednisolone	3.65±1.18	7.00±4.81	−3.35 (−5.94, −0.76)
	Overall (I^2 = 0.0%, *P* = 0.965)				−3.30 (−4.07, −2.52)
	Rana et al., 2018	Nebulized budesonide and IV methylprednisolone	3.00±1.85*	4.00±9.63*	−1.00 (−2.64, 0.64)
	Overall (I^2 = %, *P* =)				−1.00 (−2.64, 0.64)
	**Duration of mechanical ventilation (days)**				
	Wu et al., 1999	IV dexamethasone	3.50±3.10	4.60±3.00	−1.10 (−2.79, 0.59)
	Suresh et al., 2015	Nebulized budesonide	0±0	0.2±0.41	NA
	Rana et al., 2018	Nebulized budesonide and IV methylprednisolone	2.00±1.48*	4.00±1.85*	−2.00 (−2.40, −1.60)
	**Downes' score day 3**				
	Garg et al., 2016	Nebulized budesonide	1.00±1.41	2.61±1.27	−1.61 (−2.21, −1.01)
	Rana et al., 2018	Nebulized budesonide and IV methylprednisolone	1.00±2.22*	2.00±3.33*	−1.00 (−1.67, −0.33)
	**Downes' score day 5**				
	Garg et al., 2016	Nebulized budesonide	0.36±0.58	0.81±0.92)	−0.45 (−0.79, −0.11)
	Rana et al., 2018	Nebulized budesonide and IV methylprednisolone	0±1.48*	2.00±1.48*	−2.00 (−2.35, −1.65)
	**Duration of X-ray clearance (days)**				
	Yeh et al., 1977	IV hydrocortisone	4.50±0.6	4.20±0.5	0.30 (−0.07, 0.67)
	Overall (I^2 = %, *P* =)				0.30 (−0.07, 0.67)
	Basu et al., 2007	Nebulized budesonide	6.56±1.22	15.82±1.26	−9.26 (−9.86, −8.66)
	Tripathi et al., 2007	Nebulized budesonide	5.18±1.80	7.76±3.31	−2.58 (−4.54, −0.62)
	Overall (I^2 = 97.6%, *P* = 0.00)				−5.99 (−12.53, 0.56)
	Basu et al., 2007	IV methylprednisolone	6.65±1.07	15.82±1.26	−9.17 (−9.73, −8.61)
	Tripathi et al., 2007	IV methylprednisolone	5.41±1.90	7.76±3.31	−2.35 (−4.33, −0.37)
	Overall (I^2 = 97.6%, *P* = 0.00)				−5.83 (−12.51, 0.85)
	**Duration of hospitalization, NICU stay, and PICU stay (days)**				
	Wu et al., 1999	IV dexamethasone	14.00±5.00	14.00±6.00	0.00 (−3.09, 3.09)

	Overall (I^2 = %, P =)				0.00 (−3.09 to 3.09)
	Basu et al., 2007	Nebulized budesonide	10.63±1.56	18.06±2.33	−7.43 (−8.39, −6.47)
	Tripathi et al., 2007	Nebulized budesonide	12.18±6.22	19.59±12.77	−7.41 (−14.80, −0.02)
	Suresh et al., 2015	Nebulized budesonide	7.58±2.81	10.47±5.21	−2.89 (−5.67, −0.11)
	Garg et al., 2016	Nebulized budesonide	4.41±1.68	5.79±2.21	−1.38 (−2.25, −0.51)
	Overall (I^2 = 96.5%, P =0.00)				−4.47 (−8.64, −0.30)
	Basu et al., 2007	IV methylprednisolone	10.82±1.64	18.06±2.33	−7.24 (−8.21, −6.27)
	Tripathi et al., 2007	IV methylprednisolone	13.29±7.48	19.59±12.77	−6.30 (−13.99, 1.39)
	Overall (I^2 = 0.0%, P = 0.812)				−7.23 (−8.19, −6.27)
	Rana et al., 2018	Nebulized budesonide and IV methylprednisolone	8.00±1.48*	9.00±12.59*	−1.00 (−3.12, 1.12)
	Overall (I^2 = %, P =)				−1.00 (−3.12, 1.12)
	Tan et al., 2016	Intratracheal PS with budesonide	16.00±3.00	24.00±5.00	−8.00 (−9.93, −6.07)
	Overall (I^2 = %, P =)				−8.00 (−9.93, −6.07)
	**Time until achievement of full feeding (days)**				
	Basu et al., 2007	Nebulized budesonide	6.41±0.87	13.91±1.23	−7.50 (−8.02, −6.98)
	Suresh et al., 2015	Nebulized budesonide	3.79±1.62	8.76±4.97	−4.97 (−7.44, −2.50)
	Overall (I^2 = 74.1%, P = 0.050)				−6.54 (−8.94, −4.13)
	Basu et al., 2007	IV methylprednisolone	6.56±1.40	13.91±1.23	−7.35 (−7.98, −6.72)
	Overall (I^2 = %, P =)				−7.35 (−7.98, −6.72)
	**Duration of IV fluid requirement (days)**				
	Basu et al., 2007	Nebulized budesonide	5.96±1.03	12.91±1.23	−6.95 (−7.50, −6.40)
	Tripathi et al., 2007	Nebulized budesonide	NR	NR	
	Basu et al., 2007	IV methylprednisolone	5.85±1.26	12.91±1.23	−7.06 (−7.66, −6.46)
	Tripathi et al., 2007	IV methylprednisolone	NR	NR	
	**Author**	**Intervention**	**Steroids**	**Control**	**Relative risk (95% CI)**
			**Event/Total**	**Event/Total**	
	**Need for mechanical ventilation**				
**Adverse drug reactions**	Yeh et al., 1977	IV hydrocortisone	4/17	3/18	1.41 (0.37,5.40)
	Overall (I^2 = %, P =)				1.41 (0.37, 5.40)

	Sangeetha et al., 2017	IV dexamethasone	5/30	9/30	0.56 (0.21, 1.46)
	Patil et al., 2018	IV dexamethasone	4/34	1/36	4.24 (0.5, 36.02)
	Overall (I^2 = 66.5%, *P* = 0.084)				1.23 (0.17, 8.87)
	Basu et al., 2007	Nebulized budesonide	0/32	NR	NA
	Garg et al., 2016	Nebulized budesonide	12/39	10/39	1.20 (0.59, 2.45)
	Basu et al., 2007	IV methylprednisolone	0/34	NR	NA
	Tan et al., 2016	Intratracheal PS with budesonide	3/35	7/35	0.43 (0.12, 1.52)
	**Meningitis**				
	Basu et al., 2007	Nebulized budesonide	3/32	3/33	1.03 (0.22, 4.74)
	Tripathi et al., 2007	Nebulized budesonide	2/17	4/17	0.50 (0.11, 2.38)
	Suresh et al., 2015	Nebulized budesonide	1/20	1/20	1.00 (0.07, 14.90)
	Overall (I^2 = 0.0%, *P* = 0.790)				0.76 (0.28, 2.08)
	Basu et al., 2007	IV methylprednisolone	3/34	3/33	0.97 (0.21, 4.47)
	Tripathi et al., 2007	IV methylprednisolone	3/17	4/17	0.75 (0.20, 2.86)
	Overall (I^2 = 0.0%, *P* = 0.803)				0.84 (0.31, 2.29)
	**Sepsis without meningitis**				
	Patil et al., 2018	IV dexamethasone	3/34	5/36	0.64 (0.16, 2.46)
	Overall (I^2 = %, *P* =)				0.64 (0.16, 2.46)
	Basu et al., 2007	Nebulized budesonide	1/32	2/33	0.52 (0.05, 5.41)
	Tripathi et al., 2007	Nebulized budesonide	3/17	9/17	0.33 (0.11, 1.02)
	Suresh et al., 2015	Nebulized budesonide	3/20	3/20	1.00 (0.23, 4.37)
	Garg et al., 2016	Nebulized budesonide	3/39	5/39	0.60 (0.15, 2.34)
	Overall (I^2 = 0.0%, *P* = 0.705)				0.53 (0.26, 1.07)
	Basu et al., 2007	IV methylprednisolone	1/34	2/33	0.49 (0.05, 5.10)
	Tripathi et al., 2007	IV methylprednisolone	5/17	9/17	0.56 (0.23, 1.32)
	Overall (I^2 = 0.0%, *P* = 0.915)				0.55 (0.24, 1.23)
	Tan et al., 2016	Intratracheal PS with budesonide	0/35	2/35	0.20 (0.01, 4.02)
	Overall (I^2 = %, *P* =)				0.20 (0.01, 4.02)

	**Fungal infection**				
	Basu et al., 2007	Nebulized budesonide	2/32	3/33	0.69 (0.12, 3.85)
	Tripathi et al., 2007	Nebulized budesonide	0/7	2/17	0.20 (0.01, 3.88)
	Overall (I^2 = 0.0%, P = 0.475)				0.50 (0.11, 2.23)
	Basu et al., 2007	IV methylprednisolone	1/34	3/33	0.32 (0.04, 2.95)
	Tripathi et al., 2007	IV methylprednisolone	0/17	2/17	0.20 (0.01, 3.88)
	Overall (I^2 = 0.0%, P = 0.798)				0.27 (0.05, 1.60)
	**Oral thrush**				
	Basu et al., 2007	Nebulized budesonide	2/32	5/33	0.41 (0.09, 1.97)
	Tripathi et al., 2007	Nebulized budesonide	5/17	6/17	0.83 (0.31, 2.22)
	Overall (I^2 = 0.0%, P = 0.447)				0.68 (0.30, 1.57)
	Basu et al., 2007	IV methylprednisolone	3/34	5/33	0.58 (0.15, 2.24)
	Tripathi et al., 2007	IV methylprednisolone	3/17	6/17	0.50 (0.15, 1.68)
	Overall (I^2 = 0.0%, P = 0.869)				0.54 (0.22, 1.32)
	**Pneumothorax**				
	Suresh et al., 2015	Nebulized budesonide	0/20	2/20	0.20 (0.01, 3.92)
	Garg et al, 201	Nebulized budesonide	1/39	3/39	0.33 (0.04, 3.07)
	Overall (I^2 = 0.0%, P = 0.787)				0.28 (0.05, 1.64)
	Yeh et al., 1977	IV hydrocortisone	3/17	5/18	0.64 (0.18, 2.26)
	Overall (I^2 = %, P =)				0.64 (0.18, 2.26)
	Sangeetha et al., 2017	IV dexamethasone	1/30	2/30	0.50 (0.05, 5.22)
	Patil et al., 2018	IV dexamethasone	0/34	0/36	NA
	Overall (I^2 = %, P =)				0.50 (0.05, 5.22)
	**Persistent pulmonary hypertension of the newborn**				
	Basu et al., 2007	Nebulized budesonide	0/32	0/33	NA
	Garg et al., 2016	Nebulized budesonide	1/39	3/39	0.33 (0.0, 3.07)
	Basu et al., 2007	IV methylprednisolone	0/34	0/33	NA
	Tan et al., 2016	Intratracheal PS with budesonide	2/35	5/35	0.40 (0.08, 1.93)

	**Pulmonary vasodilator needed**				
	Patil et al., 2018	IV dexamethasone	3/34	4/36	0.79 (0.19, 3.29)
	**Respiratory arrest**				
	Basu et al., 2007	Nebulized budesonide	4/32	3/33	1.38 (0.33, 5.66)
	Basu et al., 2007	IV methylprednisolone	3/34	3/33	0.97 (0.21, 4.47)
	**Hypotension**				
	Basu et al., 2007	Nebulized budesonide	3/32	3/33	1.03 (0.22, 4.74)
	Suresh et al., 2015	Nebulized budesonide	2/20	3/20	0.67 (0.12, 3.57)
	Overall (I^2 = 0.0%, P = 0.706)				0.85 (0.27, 2.62)
	Basu et al., 2007	IV methylprednisolone	4/34	3/33	1.29 (0.31, 5.34)
	Overall (I^2 = %, P =)				1.29 (0.31, 5.34)
	**Hypoglycemia**				
	Basu et al., 2007	Nebulized budesonide	6/32	7/33	0.88 (0.33, 2.35)
	Suresh et al., 2015	Nebulized budesonide	3/20	3/20	1.00 (0.23, 4.37)
	Overall (I^2 = 0.0%, P = 0.891)				0.92 (0.41, 2.07)
	Basu et al., 2007)	IV methylprednisolone	8/34	7/33	1.11 (0.45, 2.71)
	Overall (I^2 = %, P =)				1.11 (0.45, 2.71)
	**Hypocalcemia**				
	Basu et al., 2007	Nebulized budesonide	2/32	4/33	0.52 (0.10, 2.62)
	Suresh et al., 2015	Nebulized budesonide	0/20	0/20	NA
	Overall (I^2 = %, P =)				0.52 (0.10, 2.62)
	Basu et al., 2007	IV methylprednisolone	4/34	4/33	0.97 (0.26, 3.56)
	Overall (I^2 = %, P =)				0.97 (0.26, 3.56)
	**Seizure**				
	Basu et al., 2007	Nebulized budesonide	0/32	3/33	0.15 (0.01, 2.74)
	Suresh et al., 2015	Nebulized budesonide	1/20	2/20	0.50 (0.05, 5.08)
	Overall (I^2 = 0.0%, P = 0.512)				0.31 (0.05, 1.92)
	Basu et al., 2007	IV methylprednisolone	1/34	3/33	0.32 (0.04, 2.95)

	Overall (I^2 = %, *P* =)				0.32 (0.04, 2.95)
	**Hyperbilirubinemia**				
	Basu et al., 2007	Nebulized budesonide	5/32	5/33	1.03 (0.33, 3.23)
	Suresh et al., 2015	Nebulized budesonide	2/20	2/20	1.00 (0.16, 6.42)
	Overall (I^2 = 0.0%, *P* = 0.978)				1.02 (0.39,2.70)
	Basu et al., 2007	IV methylprednisolone	6/34	5/33	1.16 (0.39, 3.45)
	Overall (I^2 = %, *P* =)				1.16 (0.39, 3.45)
	Patil et al., 2018	IV dexamethasone	5/34	7/36	0.76 (0.27, 2.16)
	Overall (I^2 = %, *P* =)				0.76 (0.27, 2.16)
	**Death**				
	Basu et al., 2007	Nebulized budesonide	0/32	2/33	0.21 (0.01, 4.13)
	Tripathi et al., 2007	Nebulized budesonide	2/17	3/17	0.67 (0.13, 3.50)
	Suresh et al., 2015	Nebulized budesonide	1/20	3/20	0.33 (0.04, 2.94)
	Garg et al., 2016	Nebulized budesonide	3/39	4/39	0.75 (0.18, 3.13)
	Overall (I^2 = 0.0%, *P* = 0.834)				0.55 (0.22, 1.39)
	Basu et al., 2007	IV methylprednisolone	0/34	2/33	0.19 (0.01, 3.90)
	Tripathi et al., 2007	IV methylprednisolone	2/17	3/17	0.67 (0.13, 3.50)
	Overall (I^2 = 0.0%, *P* = 0.472)				0.50 (0.12, 2.13)
	Wu et al., 1999	IV dexamethasone	2/27	1/23	1.70 (0.16, 17.60)
	Patil et al., 2018	IV dexamethasone	0/34	1/36	0.35 (0.01, 8.36)
	Overall (I^2 = 0.0%, *P* = 0.431)				0.98 (0.15, 6.41)
	Yeh et al., 1977	IV hydrocortisone	1/17	2/18	0.53 (0.05, 5.32)
	Overall (I^2 = %, *P* =)				0.53 (0.05, 5.32)
	**Anemia**				
	Basu et al., 2007	Nebulized budesonide	5/32	3/33	1.72 (0.45, 6.60)
	Basu et al., 2007	IV methylprednisolone	4/34	3/33	1.29 (0.31, 5.34)
	**Stage 2 HIE**				
	Garg et al., 2016	Nebulized budesonide	2/34	1/36	0.80 (0.23, 2.76)

	Patil et al., 2018	IV dexamethasone	4/39	5/39	2.12 (0.20, 22.30)
	**Loose motions**				
	Tripathi et al., 2007	Nebulized budesonide	0/17	2/17	0.20 (0.01, 3.88)
	Tripathi et al., 2007	IV methylprednisolone	2/17	2/17	1.00 (0.16, 6.30)

Abbreviations: CI = confidence interval; HIE = hypoxic-ischemic encephalopathy; IV = intravenous; NA = not applicable; NICU = neonatal intensive care unit; NR = not reported; PICU = pediatric intensive care unit; PS = porcine lung surfactant; SD = standard deviation

*Converted data from median (interquartile range) to mean (SD)

Supplementary Figure S1. Results of the outcomes in the systematic review and meta-analysis

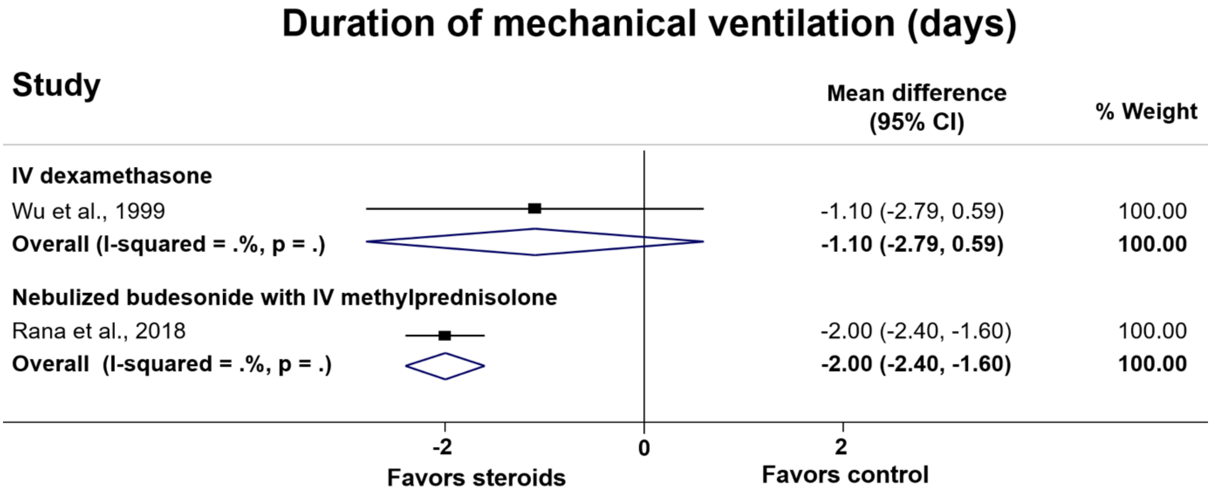

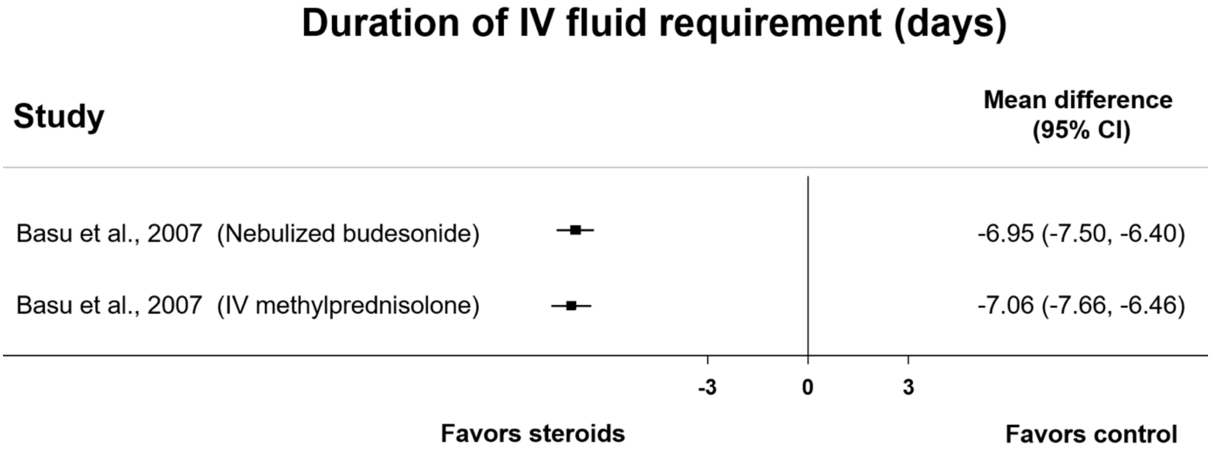

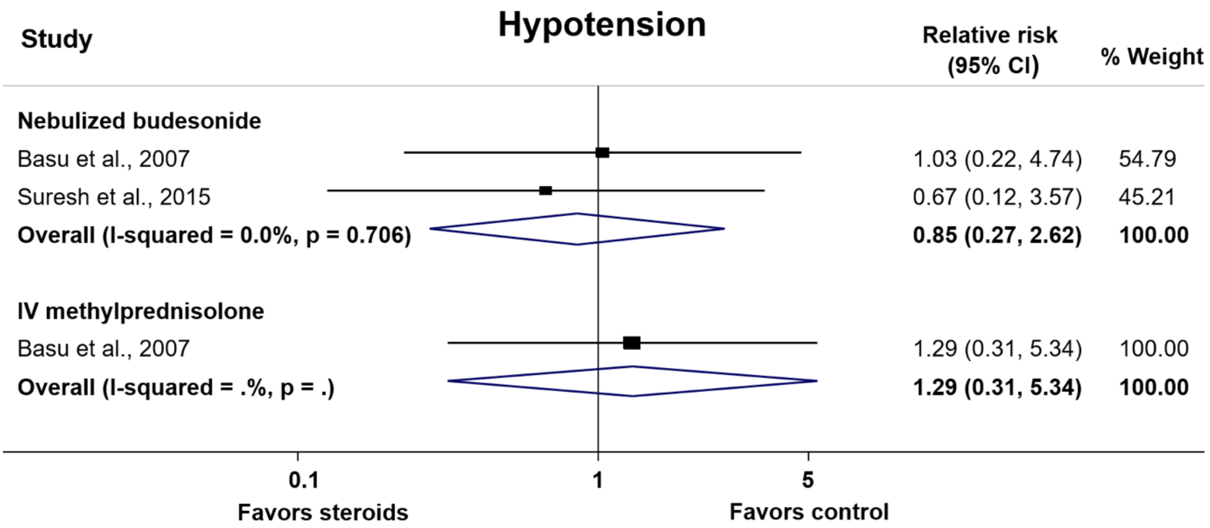

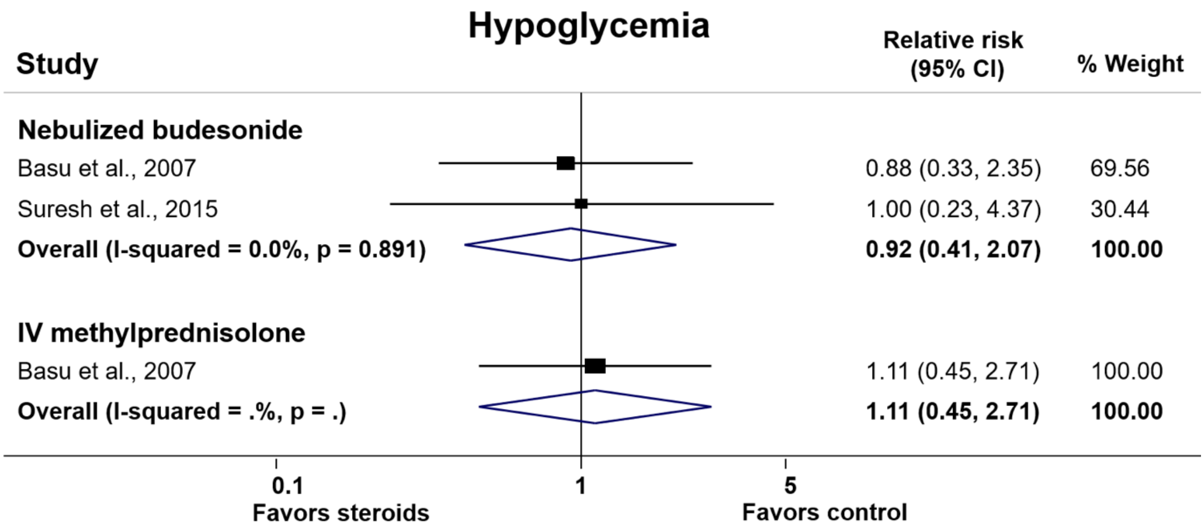
Hypoglycemia
Study
Relative risk (95% CI)
% Weight
Nebulized budesonide
Basu et al., 2007 0.88 (0.33, 2.35) 69.56
Suresh et al., 2015 1.00 (0.23, 4.37) 30.44
Overall (I-squared = 0.0%, p = 0.891) 0.92 (0.41, 2.07) 100.00
IV methylprednisolone
Basu et al., 2007 1.11 (0.45, 2.71) 100.00
Overall (I-squared = .%, p = .) 1.11 (0.45, 2.71) 100.00
0.1
1
5
Favors steroids
Favors control

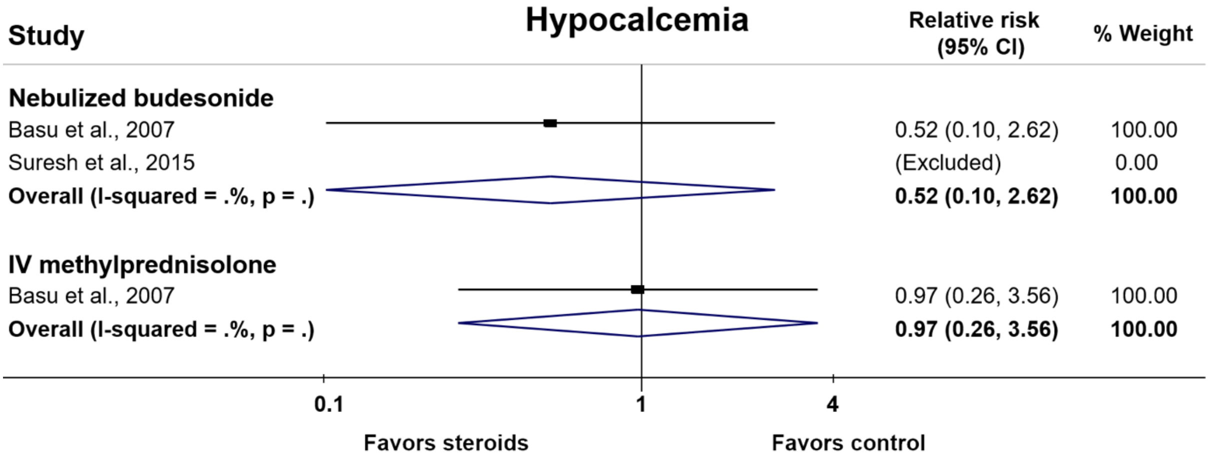
Hypocalcemia
Study
Relative risk (95% CI)
% Weight
Nebulized budesonide
Basu et al., 2007 0.52 (0.10, 2.62) 100.00
Suresh et al., 2015 (Excluded) 0.00
Overall (I-squared = .%, p = .) 0.52 (0.10, 2.62) 100.00
IV methylprednisolone
Basu et al., 2007 0.97 (0.26, 3.56) 100.00
Overall (I-squared = .%, p = .) 0.97 (0.26, 3.56) 100.00
0.1
1
4
Favors steroids
Favors control

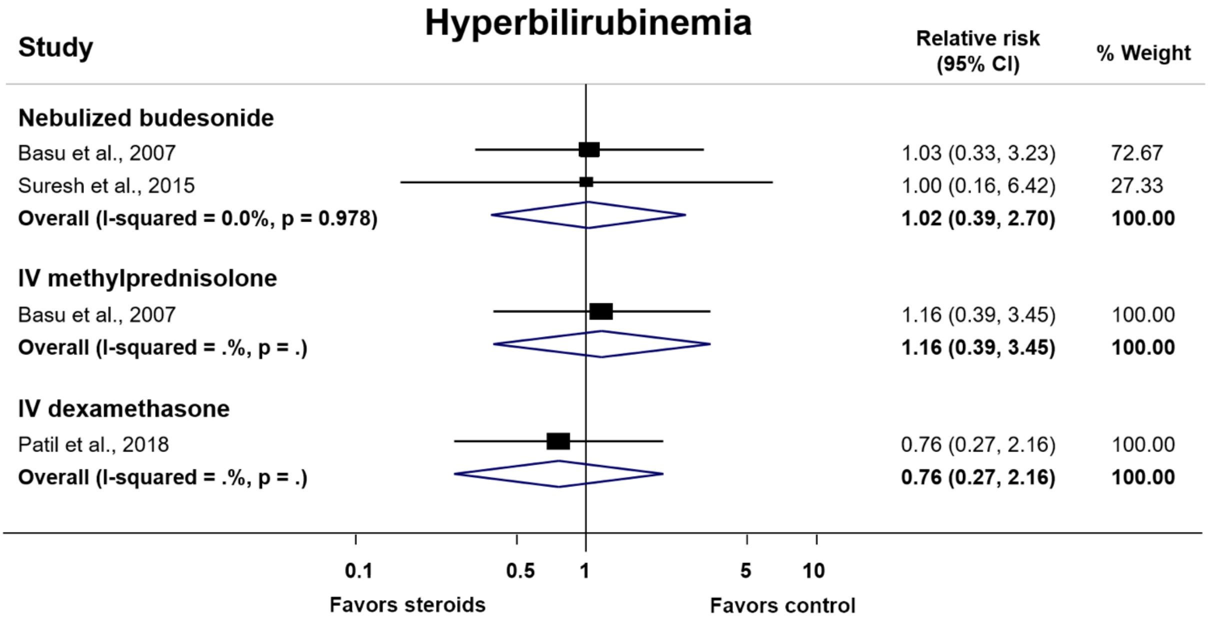
Hyperbilirubinemia
Study
Relative risk (95% CI)
% Weight
Nebulized budesonide
Basu et al., 2007 1.03 (0.33, 3.23) 72.67
Suresh et al., 2015 1.00 (0.16, 6.42) 27.33
Overall (I-squared = 0.0%, p = 0.978) 1.02 (0.39, 2.70) 100.00
IV methylprednisolone
Basu et al., 2007 1.16 (0.39, 3.45) 100.00
Overall (I-squared = .%, p = .) 1.16 (0.39, 3.45) 100.00
IV dexamethasone
Patil et al., 2018 0.76 (0.27, 2.16) 100.00
Overall (I-squared = .%, p = .) 0.76 (0.27, 2.16) 100.00
0.1
0.5
1
5
10
Favors steroids
Favors control

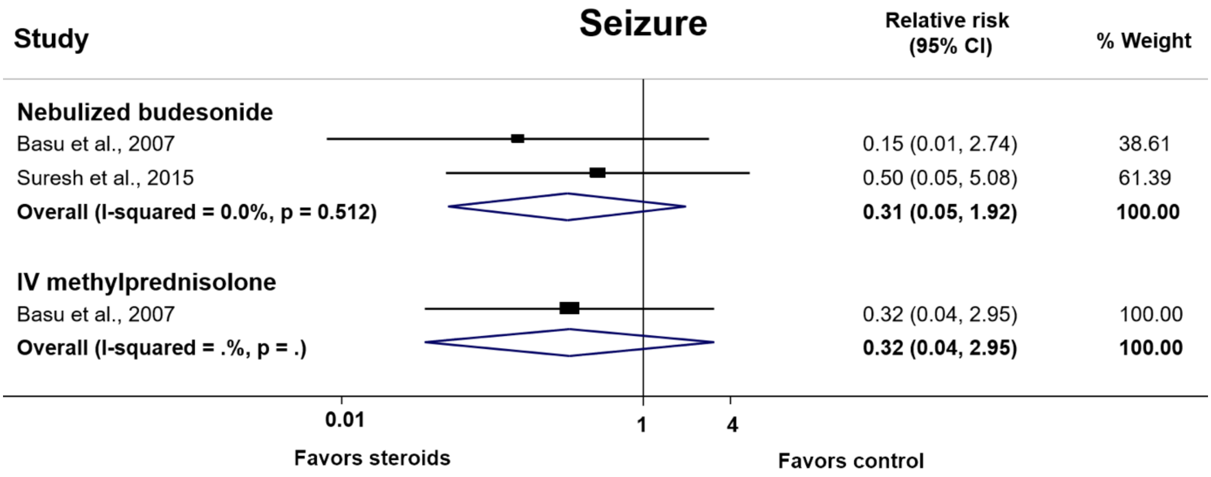
Seizure
Study
Relative risk (95% CI)
% Weight
Nebulized budesonide
Basu et al., 2007
0.15 (0.01, 2.74)
38.61
Suresh et al., 2015
0.50 (0.05, 5.08)
61.39
Overall (I-squared = 0.0%, p = 0.512)
0.31 (0.05, 1.92)
100.00
IV methylprednisolone
Basu et al., 2007
0.32 (0.04, 2.95)
100.00
Overall (I-squared = .%, p = .)
0.32 (0.04, 2.95)
100.00
0.01
1
4
Favors steroids
Favors control

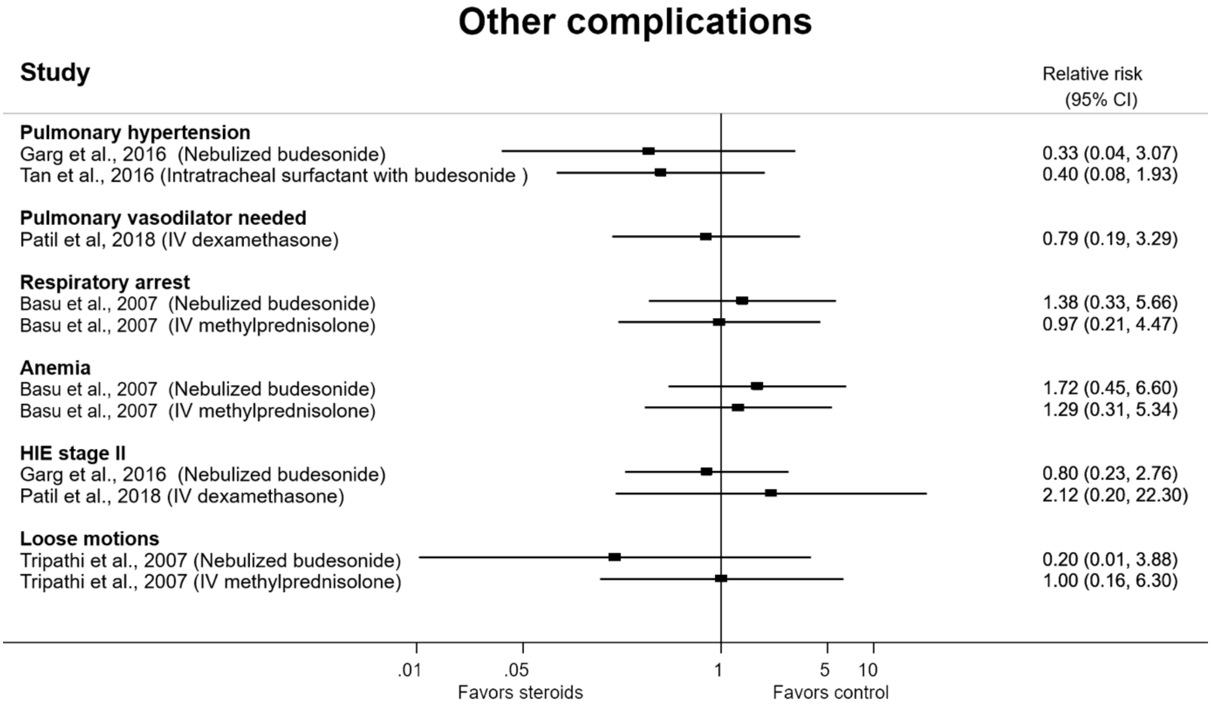
Other complications
Study
Relative risk (95% CI)
Pulmonary hypertension
Garg et al., 2016 (Nebulized budesonide)
0.33 (0.04, 3.07)
Tan et al., 2016 (Intratracheal surfactant with budesonide)
0.40 (0.08, 1.93)
Pulmonary vasodilator needed
Patil et al, 2018 (IV dexamethasone)
0.79 (0.19, 3.29)
Respiratory arrest
Basu et al., 2007 (Nebulized budesonide)
1.38 (0.33, 5.66)
Basu et al., 2007 (IV methylprednisolone)
0.97 (0.21, 4.47)
Anemia
Basu et al., 2007 (Nebulized budesonide)
1.72 (0.45, 6.60)
Basu et al., 2007 (IV methylprednisolone)
1.29 (0.31, 5.34)
HIE stage II
Garg et al., 2016 (Nebulized budesonide)
0.80 (0.23, 2.76)
Patil et al., 2018 (IV dexamethasone)
2.12 (0.20, 22.30)
Loose motions
Tripathi et al., 2007 (Nebulized budesonide)
0.20 (0.01, 3.88)
Tripathi et al., 2007 (IV methylprednisolone)
1.00 (0.16, 6.30)
.01
.05
1
5
10
Favors steroids
Favors control
